# Data on microcirculatory parameters of GTS- 21 treated rats assessed by intravital microscopy

**DOI:** 10.1016/j.dib.2017.09.045

**Published:** 2017-09-23

**Authors:** Karsten Schmidt, Sukanya Bhakdisongkhram, Florian Uhle, Christoph Philipsenburg, Aleksandar R. Zivkovic, Thorsten Brenner, Johann Motsch, Markus A. Weigand, Stefan Hofer

**Affiliations:** a Department of Anesthesiology, Heidelberg University Hospital, Im Neuenheimer Feld 110, 69120 Heidelberg, Germany; b Clinic for Anesthesiology, Intensive Care and Emergency Medicine I, Westpfalz Hospital, Hellmut-Hartert-Str. 1, 67655 Kaiserslautern, Germany

## Abstract

This article contains animal experimental data associated with the research article entitled “GTS-21 reduces microvascular permeability during experimental endotoxemia” (Schmidt et al., 2017) [1] (supplementary datasets of baseline intravital microscopic measurements, baseline TNF-α levels and vital parameters of the evaluated experimental groups are provided). Beneficial anti-inflammatory effects of cholinergic mediators on microvascular inflammation have been demonstrated by intravital microscopic investigations (Schmidt et al., 2015) [2], therefore we evaluated the effect of the cholinergic mediator GTS-21 on microcirculatory alterations during endotoxemia [1]. The data regarding microcirculatory effects of GTS-21 treatment ((3-(2,4-Dimethoxybenzylidene)-anabaseine dihydrochloride; 1 mg/kg; i.v.) in non-endotoxemic animals are presented in this article.

**Specifications Table**

**Table:** 

Subject area	*Inflammation/Immunology*
More specific subject area	*Microcirculatory alterations in sepsis*
Type of data	*figures; pictures*
How data was acquired	*Animal experiment, intravital microscopy, blood sample cytokine determination with enzyme linked immunoabsorbent assay (ELISA) for TNF-ɑ, invasive hemodynamic monitoring, temperature monitoring, blood gas analysis*
Data format	*Analyzed*
Experimental factors	*Male Wistar rats (n=10/group) were anesthetized, ventilated and surgically prepared for intravital microscopy (IVM) of mesenterial postcapillary venules; IVM included macromolecular leakage-, leukocyte adhesion- and venular wall shear rate evaluation; IVMs were performed at 60, 120 and 180 min after the baseline IVM; total experimental time was 240 min; endotoxemia induction and test substance application followed baseline IVM*
Experimental features	*IVM evaluation of GTS-21 effects on microvascular inflammation and microcirculatory alterations in endotoxemic and non-endotoxemic animals*
Data source location	*Heidelberg, Germany*
Data accessibility	*Data are available in this article*
Related research article	*Schmidt K, Bhakdisongkhram S, Uhle F, Philipsenburg C, Zivkovic AR, Brenner T, Motsch J, Weigand MA, Hofer S. GTS-21 reduces microvascular permeability during experimental endotoxemia. Microvasc Res (2017),*https://doi.org/10.1016/j.mvr.2017.08.002

**Value of the data**

• The supplementary datasets provide hemodynamic parameters and baseline microcirculatory data of an in vivo animal experimental method
• The datasets can be useful for designing comparable animal experiments
• The data provide information on endotoxemia independent microcirculatory effects of GTS-21

## 1 Data

Cholinergic mediators have been demonstrated to exert anti-inflammatory effects via the α7 nicotinic acetylcholine receptor (α7nAChR) during inflammation. The datasets provide information from animal experiments associated with the evaluation of microcirculatory effects of the partial α7nAChR GTS-21 during lipopolysaccharide (LPS) induced endotoxemia (Schmidt et al., 2017) [1].

**Fig. 1:**
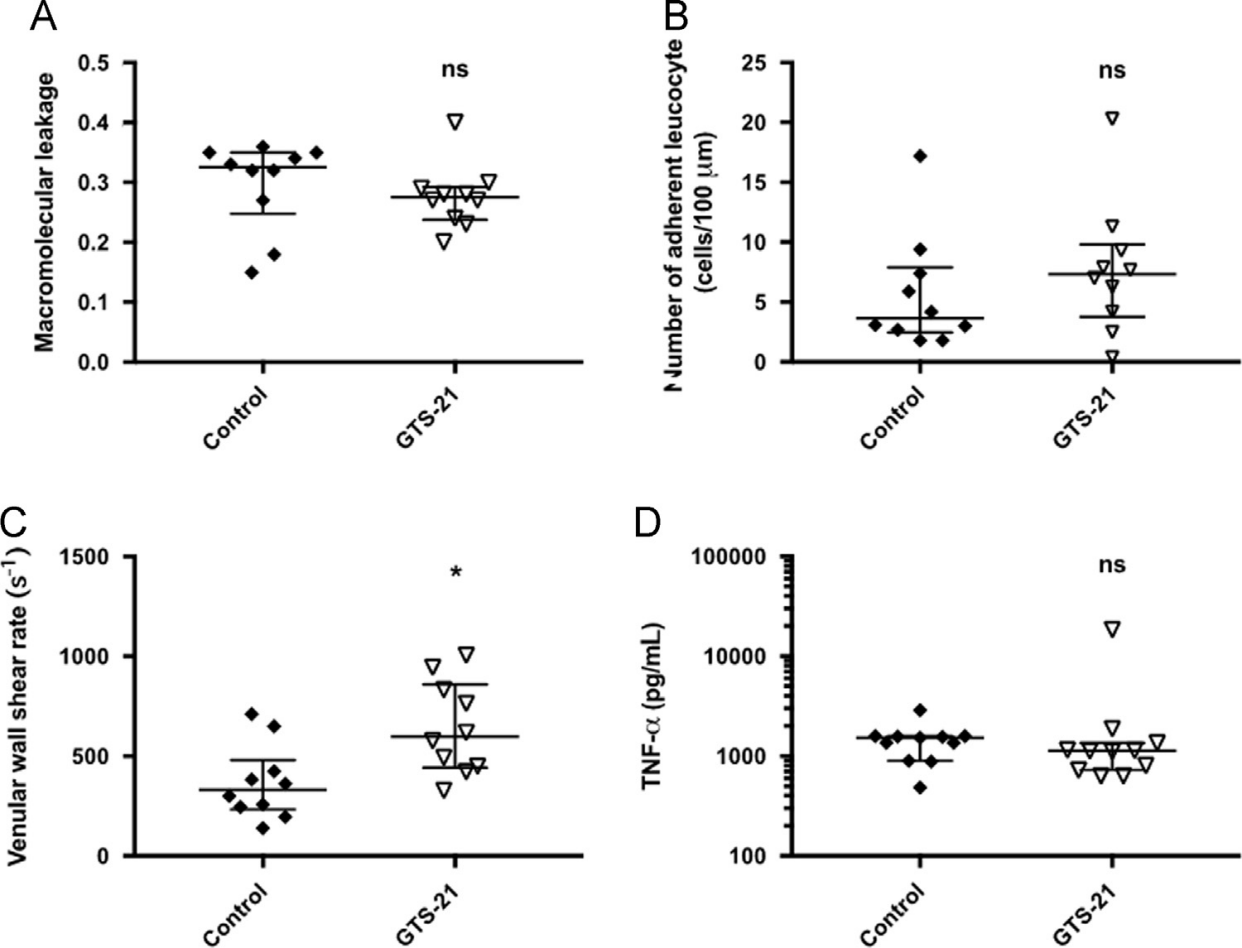
Data on microcirculatory parameters of GTS-21 treated non-endotoxemic animals compared with the crystalloid treated control group 180 min after the baseline IVM. A: No significant difference in macromolecular leakage was observed between the non-endotoxemic GTS-21 control group and the control group 180 min after baseline IVM (scatter plots with medians and interquartile range (Q1–Q3) are displayed; ns: not significant). B: No significant difference in the number of adherent leukocytes was observed between the non-endotoxemic GTS-21 control group and the control group 180 min after baseline IVM (adherent leukocytes are expressed as cells/100 µm venule length; scatter plots with medians and interquartile range (Q1–Q3) are displayed; ns: not significant). C: Venular wall shear rate was significantly increased in postcapillary venules in the non-endotoxemic GTS-21 control group compared to the control group 180 min after baseline IVM (venular wall shear rate is expressed as ^s−1^; * non-endotoxemic GTS 21 control vs. control; *p*<0.05; scatter plots with medians and interquartile range (Q1–Q3) are displayed). D: No significant difference in TNF-α levels was observed between the non-endotoxemic GTS-21 group and the control group 180 min after baseline IVM (TNF-α levels are expressed as pg/ml; scatter plots with medians and interquartile range (Q1–Q3) are displayed; ns: not significant).

Fig. 1 shows data of the non-endotoxemic GTS-21 treated experimental group compared with the crystalloid treated control group 180 min after the baseline IVM. 1 mg/kg GTS-21 was applied i.v. directly after the baseline IVM in non-endotoxemic GTS-21 treated animals to evaluate endotoxemia independent GTS-21 effects on the microcirculatory parameters.

**Fig. 2:**
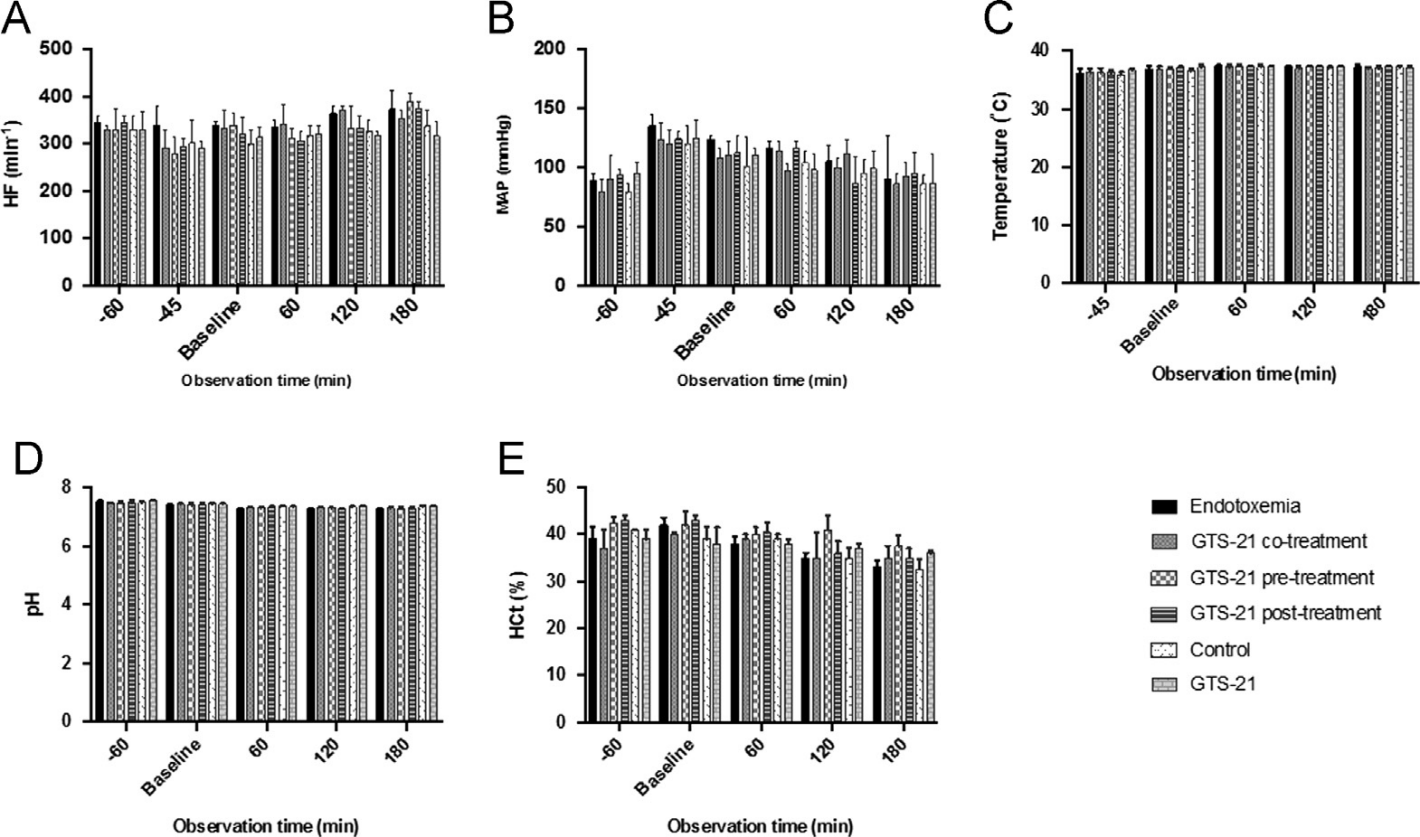
Vital parameters during the 240 min experiment in the experimental groups. A: heart frequency (HF, (min^−1^)), B: mean arterial pressure (MAP, mmHg)), C: temperature (°C), 2D: pH, E: hematocrit (HCt, (%)); bar diagrams with medians and interquartile range (Q1–Q3) are displayed.

**Fig. 3:**
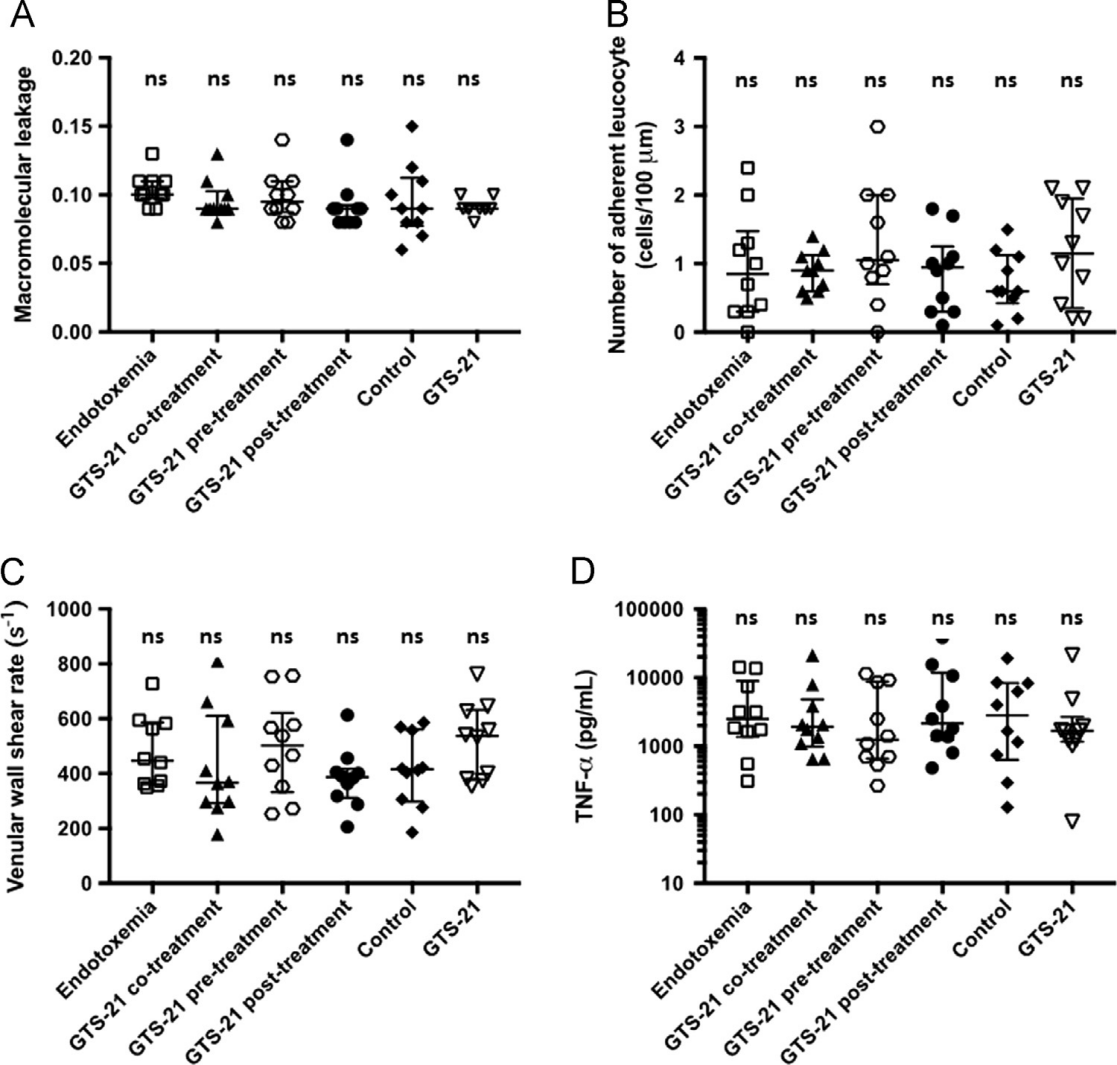
Microcirculatory parameters and TNF α levels at the baseline IVM. No significant differences were observed between all experimental groups (*n*=10/group) at the baseline IVM before randomization (scatter plots with medians and interquartile range (Q1–Q3) are displayed; ns: not significant). A: Macromolecular leakage of fluorescein isothiocyanate-labeled bovine albumin expressed as the ratio of perivenular to venular fluorescence intensity in arbitrary units. B: Number of adherent leukocytes expressed as cells/100 µm venule length. C: Venular wall shear rate based on mean red blood cell velocities expressed as s^−1^. D: TNF-α levels expressed as pg/ml.

**Fig. 4:**
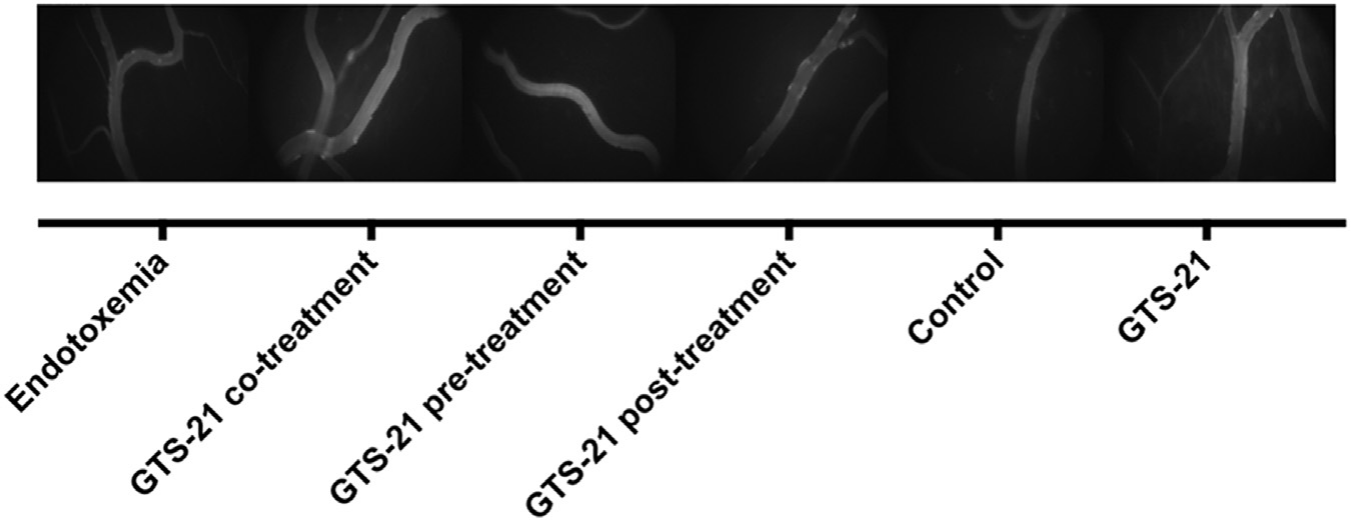
Exemplary fluorescent IVM images of postcapillary venules with the approximate median perivenular macromolecular leakage values of the respective experimental groups at the baseline IVM visualizing the results of Fig. 3A.

Fig. 2 provides descriptive data of the evaluated vital parameters during the 240-min experiment in the respective experimental groups. Fig. 3 demonstrates the results of the baseline IVM and baseline systemic TNF-ɑ levels before randomization to the respective experimental groups; all animals were treated identically until completion of this baseline IVM. Fig. 4 displays exemplary intravital fluorescent images of postcapillary venules and their perivenular macromolecular leakage at baseline IVM. The exemplary images correspond to the data presented in Fig. 3A and visualize comparable perivenular macromolecular leakage in all groups before randomization.

## 2 Experimental design, materials and methods

All experimental procedures were performed under the German legislation on animal protection and approved by the Governmental Animal Protection Committee (Project licence no. 35-9185.81/G-185/15). The experimental design, methods and chemical reagents are presented in detail in Ref. [1]:

In brief, male Wistar rats (*n*=60) were anesthetized, ventilated and surgically prepared for IVM evaluation of macromolecular leakage, leukocyte adhesion and venular wall shear rate in postcapillary mesenterial venules. Vital parameters were controlled throughout the total experimental time of 240 min (Fig. 2). The experimental protocol comprised two timepoints for the i.v. application of the test reagents (LPS (5 mg/kg), GTS-21 (1 mg/kg), crystalloid solution): the first timepoint t0 was after the baseline IVM and t1 followed 60 min later upon completion of the second IVM. All animals were treated identically until completion of the baseline IVM (Fig. 3, Fig. 4) and were randomized afterwards to treatment groups (*n*=10/group): endotoxemia group with LPS at t0; co-treatment group with GTS-21 and LPS at t0; pre-treatment group with GTS-21 at t0 and LPS at t1; post-treatment group with LPS at t0 and GTS-21 at t1; control group with crystalloid solution at t0; non-endotoxemic GTS-21 group with GTS-21 at t0. The experimental protocol was designed to evaluate GTS-21 effects in endotoxemic GTS-21 treated animals compared to untreated endotoxemic animals 180 min after the baseline IVM. Endotoxemia independent GTS-21 effects within our experimental model were evaluated by comparing the non-endotoxemic GTS-21 group with the control group 180 min after the baseline IVM (Fig. 1).

Intravital microscopy equipment, picture acquisition and evaluation criteria have been described in detail in Ref. [2]. For the quantification of perivenular macromolecular leakage, 50 mg/kg of FITC-albumin was injected 10 min before baseline IVM. IVM images were digitized and the gray levels reflecting fluorescent intensity were measured within the venule under study (iv) as well as in an equal and contiguous area of the perivenular interstitium (ii). Macromolecular leakage was determined as the ii/iv ratio. Transillumination microscopy was used to evaluate adherent leukocyte (defined as cells that did not move or detach from the endothelial wall for 30 s). Venular wall shear rate was calculated by the Newtonian definition (*γ*=8 (VRBC/Dv)), using measured vessel diameters (Dv) and the mean red blood cell velocities (VRBC) of 20–30 individual fluorescent-labeled erythrocytes. The systemic inflammatory response was evaluated by measuring TNF-ɑ levels after the baseline IVM and the 180 min IVM (Rat TNF-α ELISA MAXTM Deluxe Sets, BioLegend, San Diego, CA, USA).

## 3 Statistical analysis

Data are expressed as median with interquartile range (Q1–Q3) unless otherwise noted. The D'Agostino-Pearson omnibus normality test was applied to check for normal distribution. Due to non-normally distributed data, nonparametric methods for evaluation were used [1]. Data were considered statistically significant at *p*<0.05. All statistical analyses were performed using GraphPad Prism 7 for Mac OS X (GraphPad Software, La Jolla, CA, USA).

## Funding

This work was supported by the B. Braun-Stiftung, Melsungen, Germany.
