# Supplementary material for: Data on microcirculatory parameters of GTS- 21 treated rats assessed by intravital microscopy
Source: Data Brief. 2017 Sep 23;15:228–33. doi: 10.1016/j.dib.2017.09.045 (PMC5633251; doi:10.1016/j.dib.2017.09.045)
Supplement: Supplementary file 1 — Transparency document [file mmc1.docx]

**Cover letter/Conflict of interest declaration**

***Data in Brief* article; DIB-D-17-00746**

**Title:** ***Data on microcirculatory effects of GTS-21 assessed by intravital microscopy and presentation of vital parameters and baseline microcirculatory parameters of an animal endotoxemia model***

**Authors/Affiliations:**

Karsten Schmidt, MD*, Sukanya Bhakdisongkhram, MD*, Florian Uhle, PhD*, Christoph Philipsenburg, MD*, Aleksandar R. Zivkovic, MD*, Thorsten Brenner, MD*, Johann Motsch, MD*, Markus A. Weigand*, MD, Stefan Hofer, MD^†^

*Department of Anesthesiology, Heidelberg University Hospital

Im Neuenheimer Feld 110, 69120 Heidelberg, Germany

^†^Clinic for Anesthesiology, Intensive Care and Emergency Medicine I, Westpfalz Hospital, Kaiserslautern, Germany

Heidelberg, 18.08.2017

Dear Editor in Chief,

on behalf of all the authors, I would like to submit the enclosed manuscript entitled *“Data on microcirculatory effects of GTS-21 assessed by intravital microscopy and presentation of vital parameters and baseline microcirculatory parameters of an animal endotoxemia model”* for publication in *Data in Brief*. We thank you for considering this manuscript as a companion *Data in brief* article to our research article “*GTS-21 reduces microvascular permeability during experimental endotoxemia*” (Schmidt, K et al. GTS-21 reduces microvascular permeability during experimental endotoxemia. Microvascular Research (2017), DOI information: 10.1016/j.mvr.2017.08.002), that has been accepted for publication in *Microvascular Research*. The manuscript has been approved by all authors. It has not been published and is not under consideration for publication elsewhere in whole or in part in any language. Any potential conflicts of interest arising from associations with commercial or corporate interests regarding the manuscript are disclosed. We hope you will find the manuscript appropriate for publication in *Data in Brief.*

On behalf of all the authors

Sincerely yours,

Karsten Schmidt, MD

Corresponding author

**Declaration of interest by the authors:**

Conflict of interest: none. All authors read and approved the final manuscript.

Karsten Schmidt, MD	Sukanya Bhakdisongkhram, MD

Christoph Philipsenburg, MD	Thorsten Brenner, MD

Florian Uhle, PhD	Aleksandar Zivkovic, MD

Johann Motsch, MD	Markus A. Weigand, MD

Stefan Hofer, MD
